# Identification of Gastric Cancer-Related Circular RNA through Microarray Analysis and Bioinformatics Analysis

**DOI:** 10.1155/2018/2381680

**Published:** 2018-03-18

**Authors:** Wei Gu, Ying Sun, Xiong Zheng, Jin Ma, Xiao-Ying Hu, Tian Gao, Mei-Jie Hu

**Affiliations:** ^1^Department of Gastroenterology, Ruijin Hospital, Luwan Branch, Shanghai Jiaotong University School of Medicine, Shanghai, China; ^2^Department of Geriatrics, Renji Hospital, Shanghai Jiaotong University School of Medicine, Shanghai, China

## Abstract

Gastric cancer is one of the common malignant tumors worldwide. Increasing studies have indicated that circular RNAs (circRNAs) play critical roles in the cancer progression and have shown great potential as useful markers and therapeutic targets. However, the precise mechanism and functions of most circRNAs are still unknown in gastric cancer. In the present study, we performed a microarray analysis to detect circRNA expression changes between tumor samples and adjacent nontumor samples. The miRNA expression profiles were obtained from the National Center of Biotechnology Information Gene Expression Omnibus (GEO). The differentially expressed circRNAs and miRNAs were identified through fold change filtering. The interactions between circRNAs and miRNAs were predicted by Arraystar's home-made miRNA target prediction software. After circRNA-related miRNAs and dysregulated miRNAs were intersected, 23 miRNAs were selected. The target mRNAs of miRNAs were predicted by TarBase v7.0. Gene ontology (GO) enrichment analysis and pathway analysis were performed using standard enrichment computational methods for the target mRNAs. The results of pathway analysis showed that p53 signaling pathway and hippo signal pathway were significantly enriched and CCND2 was a cross-talk gene associated with them. Finally, a circRNA-miRNA-mRNA regulation network was constructed based on the gene expression profiles and bioinformatics analysis results to identify hub genes and hsa_circRNA_101504 played a central role in the network.

## 1. Introduction

Gastric cancer is one of the common malignant tumors in the clinic, with the second leading cause of cancer-related death worldwide [1, 2]. Although the treatment of gastric cancer has gradually improved, cure rate of gastric cancer is still low. With a lack of obvious clinical symptoms at early stage, most patients have lost the opportunity of surgical therapy when gastric cancer is detected at advanced stage [3]. Recurrence is the chief cause of gastric cancer-related death. According to recent statistics, more than 30% of patients suffering from stage III gastric cancer who undergo surgical resection develop recurrence or distant metastasis with a 14-month median recurrence-free survival time [4, 5]. Therefore, prevention and cure of gastric cancer are still challenged in the clinic and the search for new molecular markers to monitor and intervene in gastric cancer carcinogenesis is urgent.

Recent research has shown that circular RNAs (circRNAs) play a critical role in the initiation and progression of human diseases especially in tumors and may function as potential molecular markers for disease diagnosis and treatment [6]. Circular RNAs are a class of endogenous noncoding RNAs, characterized by their covalently closed-loop structures without a 5′ cap or a 3′ Poly(A) tail. Previous studies demonstrated that circRNAs existed widely in all kinds of organizations [7–9] and played a strong regulatory function in cancer [10, 11]. For example, circRNAs are dysregulated in epithelial tumors, such as laryngeal cancer and digestive system cancers [12–14], and in stromal tumors, such as gliomas [15]. Compared with mRNAs, circRNAs are more stable due to the existence of ring structure. Thus, circRNA can serves as a convenient tool for qRT-PCR measurements in cancer.

CircRNAs have been investigated for more than 40 years [16], but they have been considered as a result of splicing errors for several decades and their biological functions are largely unknown. With the development of RNA sequencing (RNA-seq) technologies and bioinformatics, circRNAs have been extensively explored in recent years and several functions of circRNA have been revealed, such as acting as scaffolds in the assembly of protein complexes [17], sequestering proteins from their native subcellular localization [18], modulating the expression of parental genes [19], regulating alternative splicing [20] and RNA-protein interactions [21], and functioning as microRNA (miRNA) sponges [8].

Our study aimed to establish the expression profile of gastric cancer through circRNA microarray chip detection. Our results revealed the potential role of circRNAs in gastric cancer. We also aimed to identify the hub circRNAs involved in gastric cancer through bioinformatics analysis. The miRNA expression profiles from the National Center of Biotechnology Information Gene Expression Omnibus (GEO) were used to identify circRNA-related dysregulated miRNAs in gastric cancer. Gene ontology (GO) enrichment analysis and pathway analysis revealed the potential biology function of miRNA target genes. Finally, a circRNA-miRNA-mRNA regulation network was constructed to selected hub genes and we found that hsa_circRNA_101504 played a central role in the network.

## 2. Methods

### 2.1. Clinical Samples

Six pairs of tumor and adjacent nontumor tissues were obtained from patients with gastric cancer who underwent surgery at the Ruijin Hospital, Shanghai Jiaotong University School of Medicine, between May 2011 and May 2014. None of the patients had received neoadjuvant therapy, and the samples were pathologically confirmed postoperatively as gastric cancer. The samples were taken within 10 min after tumor excision, immediately immersed in RNAlater® stabilization solution (Thermo Fisher Scientific, Carlsbad, CA, USA) and then stored at −80°C until being used in the experiments. The study was performed in accordance with the ethical standards of the Declaration of Helsinki and was approved by the Ethics Committee of Ruijin Hospital. Informed consent was obtained from all patients participating in the present study.

### 2.2. circRNA Microarray Analysis

Total RNA from each sample was quantified using the NanoDrop ND-1000. The sample preparation and microarray hybridization were performed based on Arraystar's standard protocols. Briefly, total RNA from each sample was amplified and transcribed into fluorescent cRNA utilizing random primer according to Arraystar's Super RNA Labeling protocol (Arraystar Inc.). The labeled cRNAs were hybridized onto the Arraystar Human circRNA Array (6x7K, Arraystar). After having washed the slides, the arrays were scanned by the Axon GenePix 4000B microarray scanner. Scanned images were then imported into GenePix Pro 6.0 software (Axon) for grid alignment and data extraction. Quantile normalization and subsequent data processing were performed using the R software package. Differentially expressed circRNAs with statistical significance between two groups were identified through volcano plot filtering. Differentially expressed circRNAs between two samples were identified through fold change filtering. Hierarchical clustering was performed to show the distinguishable circRNAs expression pattern among samples.

### 2.3. Annotation for circRNA/miRNA Interaction

Recent evidences have demonstrated that circular RNAs play a crucial role in fine tuning the level of miRNA mediated regulation of gene expression by sequestering the miRNAs. Their interaction with disease associated miRNAs indicates that circRNAs are important for disease regulation. The circRNA/microRNA interaction was predicted with Arraystar's home-made miRNA target prediction software based on TargetScan [22] and miRanda [23], and the differentially expressed circRNAs within all the comparisons were annotated in detail with the circRNA-miRNA interaction information.

### 2.4. miRNA Datasets and Data Analysis

The original miRNA expression profile of GSE23739 used in the present study was downloaded from the National Center of Biotechnology Information Gene Expression Omnibus (GEO). MicroRNA expression of twenty pairs of tissue samples collected from patients diagnosed with gastric cancer was determined by miRNA microarrays (platform was GPL19071) in this data. Each pair included resected primary tumor and corresponding healthy gastric mucosa. There were no replicates. Differentially expressed miRNAs were identified by using GEO2R. The target genes of differentially expressed miRNAs were predicted by using TarBase v7.0 [24] with a prediction score ≥0.8 and all the miRNA-mRNA interactions were experimentally supported.

### 2.5. Gene Function Analysis

Gene ontology (GO) enrichment analysis of miRNA target genes was implemented with DAVID (http://david.abcc.ncifcrf.gov/). GO terms (molecular function, biological processes, and cellular components) with *P* value less than 0.05 were considered significantly enriched by differential expressed genes. Kyoto Encyclopedia of Genes and Genomes (KEGG) is a database resource for understanding high-level functions and effects of the biological system (http://www.genome.jp/kegg/). DAVID was also used to test the statistical enrichment of genes or target genes of miRNA with differential expression in KEGG pathways. The networks of the pathways and pathway-related genes were constructed by using Cytoscape (version 3.4.0) plugin ClueGO [25] + Cluepedia [26] app.

### 2.6. Construction of the circRNA-miRNA-mRNA Regulation Network

Significantly expressed circRNAs and miRNAs and predicted mRNAs were superimposed onto the circRNA-miRNA-mRNA network. The network was constructed by using Cytoscape (version 3.4.0) and the network topology was analyzed by using CentiScaPe app [27].

### 2.7. Statistical Analysis

Statistical analysis was performed using SPSS 22.0 (Chicago, IL, USA). Significant differential expression levels of circRNAs or miRNAs were analyzed by Student's *t*-test and FDR filtering was used for comparative analysis. The *P* value ≤ 0.05 and absolute fold change ≥2.0 were considered statistically significant.

## 3. Results

### 3.1. Screening of Differentially Expressed circRNAs and miRNAs

**Figure 1:**
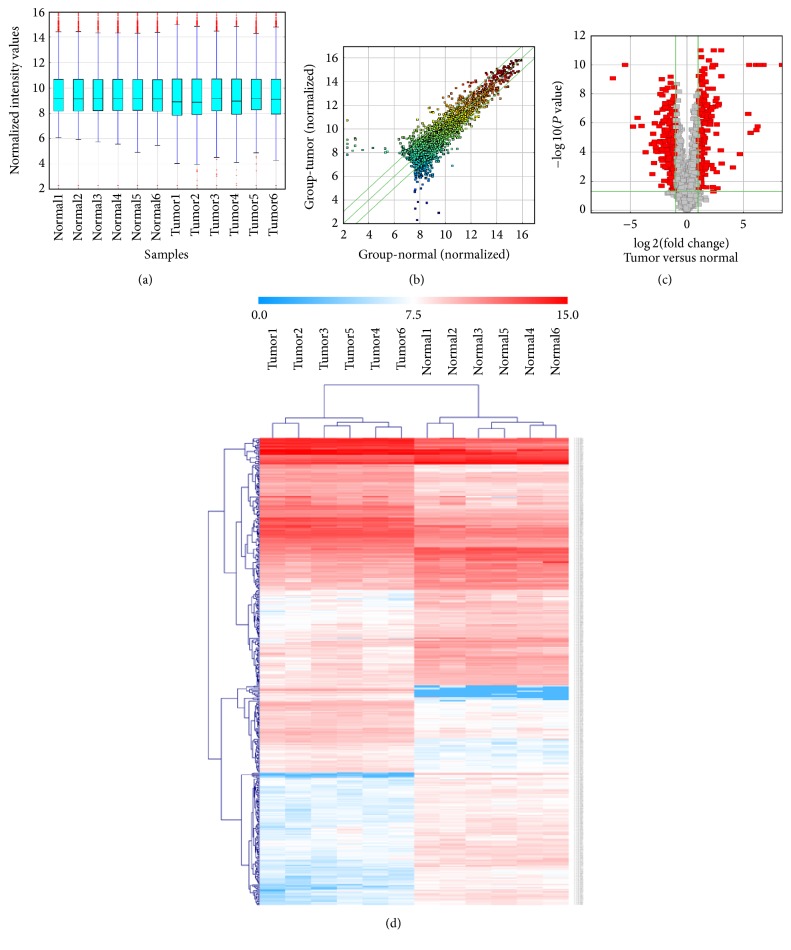
Differentially expressed circRNAs in tumor tissues and adjacent nontumor tissues from gastric cancer patients. The box plot shows the variations in circRNA expression (a). The scatter plot (b) and the volcano plot (c) illustrate the distributions of the data in the circRNA profiles. The result from hierarchical clustering shows a distinguishable circRNA expression profiling among samples. The heat map shows the differentially expressed circRNAs in tumor and adjacent nontumor tissues (d). Each group consists of six samples. Gene expression profiles are shown in rows. “Red” indicates high relative expression, and “blue” indicates low relative expression.

**Figure 2:**
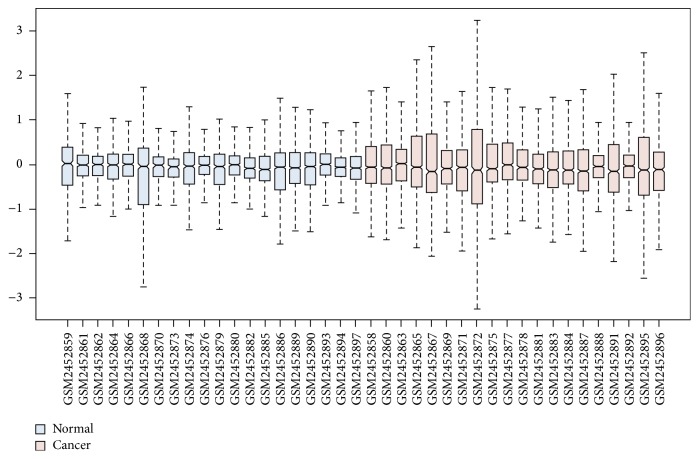
Differentially expressed miRNAs in tumor tissues and adjacent normal tissues from gastric cancer patients. The box plot shows the variations in miRNA expression. Each group consists of twenty samples.

Expression profiling data of 2070 circRNAs were obtained by using circRNA microarray analysis. The circRNA expression levels were normalized to the same order of magnitude prior to the statistical analysis. As shown in a box plot (Figure 1(a)), the median of different samples was almost on the same line after normalization, which showed a great degree of standardization. The scatter plot was used to assess the circRNA expression variation between the two compared groups of samples (Figure 1(b)). With a threshold of *P* value ≤ 0.05 and absolute value of fold change ≥2.0, a total of 440 differentially expressed circRNAs (176 significantly upregulated circRNAs and 264 significantly downregulated circRNAs) were screened (Table S1). Volcano plot was used to visualize differential expression between tumor group and adjacent nontumor group (Figure 1(c)). Hierarchical clustering was performed based on differentially expressed circRNAs to hypothesize the relationships between samples and the result of hierarchical clustering showed a distinguishable circRNA expression profiling among samples (Figure 1(d)). The miRNA expression profile of GSE23739 was analyzed by using the online tool GEO2R. The box plot showed a great degree of standardization (Figure 2). With a threshold of *P* value ≤ 0.05 and absolute value of fold change ≥2.0, a total of 111 differentially expressed miRNAs including 20 upregulated miRNAs and 91 downregulated miRNAs were identified (Table S2).

### 3.2. Prediction of circRNA-miRNA and miRNA-mRNA Interaction

**Figure 3:**
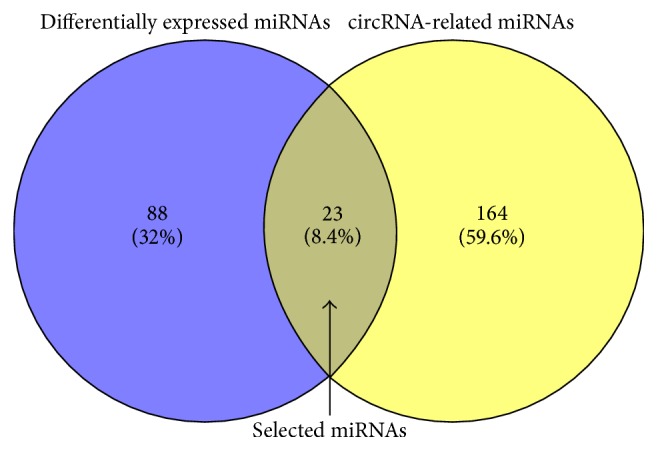
Based on differentially expressed miRNAs and circRNA-related miRNAs, the overlapped 23 miRNAs were selected using Venn graphing.

Differentially expressed circRNAs contain corresponding miRNA binding sites. To facilitate the investigation, the interactions between miRNAs and circRNAs were predicted by Arraystar's home-made miRNA target prediction software. The circRNAs with an absolute value of fold change ≥5.0 were selected for further analysis and 260 interactions between 53 circRNAs and 187 miRNAs were screened. 23 miRNAs were selected after differentially expressed miRNAs and circRNA-related miRNAs were intersected (Figure 3). The target genes of the 23 miRNAs were predicted by using TarBase v7.0 and 206 interactions between the 23 miRNAs and 150 mRNAs were obtained.

### 3.3. The GO and KEGG Enrichment Analysis of the Target Genes

**Figure 4:**
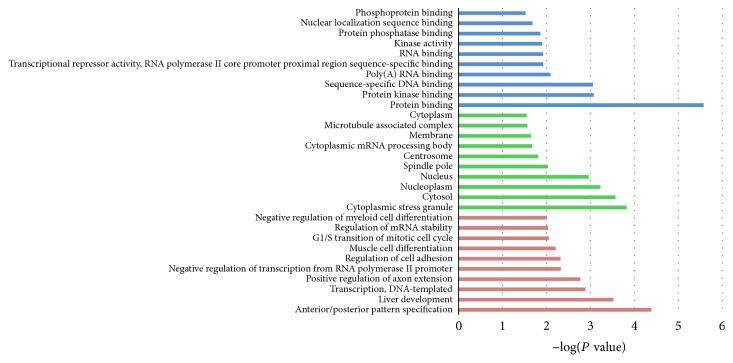
The top 10 enrichment scores in gene ontology (GO) enrichment analysis on target genes of selected miRNAs. Green bars represent cell component terms. Blue bars represent molecular function terms.

**Figure 5:**
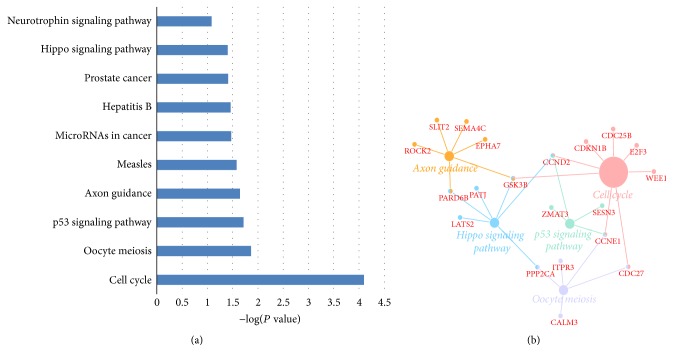
The KEGG pathway enrichment analysis on target genes of the selected miRNAs. (a) The top 10 enrichment scores in the KEGG pathway analysis of the target genes are shown. (b) The network composed of the most enriched pathways and their related genes is shown.

GO and KEGG enrichment analysis were performed for the selected 150 mRNAs to investigate the biological function of the circRNAs. In GO analysis, all the results were ranked by enrichment score (−log⁡(*P* value)) and top 10 of every category were displayed in Figure 4. In the biological process analysis, anterior/posterior pattern specification, liver development and transcription, and DNA-templated were the top 3 enriched terms. In the cellular component analysis, cytoplasmic stress granule, cytosol, and nucleoplasm were the top 3 enriched terms. In the molecular function analysis, protein binding, protein kinase binding, and sequence-specific DNA binding were the top 3 enriched terms. Results of KEGG pathway analysis were also ranked by enrichment score and the top 10 pathways associated with the mRNAs were listed in Figure 5(a). The network composed of the most enriched pathways and their related genes (Figure 5(b)) showed that PARD6B, GSK3B, CCND2, CCNE1, PPP2CA, and CDC27 were cross-talk genes associated with at least two pathways.

### 3.4. Construction of the circRNA-miRNA-mRNA Regulation Network

**Figure 6:**
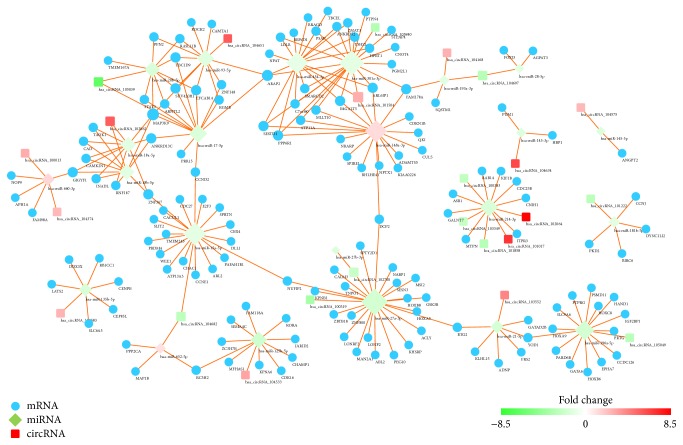
The visualization of the circRNA-miRNA-mRNA regulation network. The circular blue nodes represent mRNAs, the diamond nodes represent the miRNAs, and round rectangle nodes represent the circRNAs. “Red” indicates high relative expression, and “green” indicates low relative expression.

A circRNA-miRNA-mRNA network was constructed to reveal the interactions in circRNA, miRNA, and mRNA. As shown in Figure 6, hsa-miR-27a-3p had the most degrees and has_circRNA_101504 had the most interactions with miRNAs, indicating that they were hub genes in the regulation network. Dramatically, hsa-miR-93-5p and hsa-miR-20b-5p and hsa-miR-454-3p and hsa-miR-301a-3p were coupled miRNAs which had almost the same target genes. These coupled miRNAs might coregulate the target genes in the network. In graph theory, betweenness centrality is a measure of centrality in a graph based on shortest paths and devised as a general measure of centrality. A node with higher betweenness centrality would have more control over the network, because more information will pass through that node. The DEGs involved in the PPI network (betweenness > 4000) were listed in Table 1.

## 4. Discussion

Gastric cancer is one of the deadliest solid tumors characterized by complex molecular and cellular heterogeneity. Over the past few decades, great efforts have been made to provide novel insights into the molecular mechanisms underlying gastric cancer, but the focus has been on protein-coding genes or miRNAs [28, 29]. Recently, circRNAs has been widely reported to participate in a wide range of biological processes and their dysregulated expression is associated with many complicated human disease phenotypes including cancers [30, 31].

In this study, microarray analysis was performed to obtain the expression profiles of circRNAs in gastric cancer samples and nonmalignant pancreas samples. The expression profiles of miRNAs were obtained from GEO databases and analyzed by using GEO2R. With a threshold of *P* value < 0.05 and absolute fold change ≥2.0, dysregulated circRNAs and miRNAs were identified separately. After the circRNA-related miRNAs dysregulated miRNAs were intersected, 23 miRNAs were selected for further study. Gene function analysis including GO analysis and KEGG pathway analysis was conducted for the target mRNAs of the selected miRNAs. The results of KEGG pathway analysis indicated that p53 signaling pathway and hippo signaling pathway were significantly enriched. P53 is a well-known tumor suppressor gene and the p53 mutations have been reported in many cancers [32, 33]. In gastric cancer, He et al. [34] found that Fra-1 was upregulated in gastric cancer tissues and played its function by affecting the PI3K/Akt and p53 signaling pathway. Hippo signaling pathway is a newly discovered and conserved signaling cascade, first identified in drosophila [35]. Hippo signal pathway regulates organ size control by governing cell proliferation and apoptosis and is reported to be a tumor-suppressive signal pathway. As shown in Figure 5(b), CCND2 is an important cross-talk gene associated with cell cycle, p53 signaling pathway, and hippo signal pathway. CCND2 also has a high betweenness centrality in the PPI network, indicating that CCND2 might be a bridge of a lot of interactions. For example, CCND2 is a bridge of the target genes of hsa-miR-15a-5p and hsa-miR-93-5p. Zhang et al. [36] have reported that miR-206 could inhibit gastric cancer proliferation in part by repressing CCND2. Meanwhile, another study showed that dysregulation of miR-206-CCND2 axis might contribute to the aggressive progression and poor prognosis of human gastric cancer in clinical settings. Combined detection of their expression might be particularly helpful for surveillance of disease progression and treatment stratification [37]. However, the relationship between circRNA and CCND2 is still unknown. In the circRNA-miRNA-mRNA regulation network (Figure 6), we revealed that CCND2 might be regulated by hsa_circRNA_105039 and hsa_cirRNA_104682 through hsa-miR-15a-5p and hsa_circRNA_105039 separately. We also found that hsa_circRNA_101504 played a central role in the regulation network. As circRNAs can serve as a competitive endogenous RNA (ceRNA) to sponge miRNAs to regulate the target mRNAs [38, 39], upregulation of hsa_circRNA_101504 might affect several mRNAs by downregulating hsa-miR-454-3p and hsa-miR-301a-3p. In chondrosarcoma, increasing hsa-miR-454-3p can downregulate Stat3 and Atg12 to inhibit chondrosarcoma growth [40]. But, in human glioma, hsa-miR-454-3p has the opposite effect that the prognosis of glioma with high hsa-miR-454-3p expression is significantly worse compared with that of glioma with low hsa-miR-454-3p expression [41]. Therefore, more studies of hsa-miR-454-3p involved in gastric cancer are needed.

## 5. Conclusion

In conclusion, we have screened several dysregulated circRNAs through microarray analysis and annotated their function in gastric cancer by bioinformatics analysis. We will gather more clinical samples and validate our findings in future work.

## Figures and Tables

**Table 1:** The list of differentially expressed genes involved in the PPI network (betweenness > 4000).

Gene_name	Betweenness	Degree	Stress	Closeness
hsa-miR-27a-3p	13195.50	24	46060	0.0015
hsa-miR-15a-5p	12039.50	19	30488	0.0015
NUFIP2	7258.59	2	1440	0.0015
hsa-miR-148a-3p	7016.86	18	67106	0.0013
hsa-miR-17-5p	6342.49	13	156	0.0014
BTG2	6116.00	2	160	0.0013
hsa-miR-21-5p	6060.00	7	42	0.0011
DCP2	5609.67	2	5090	0.0014
ARAP2	5387.24	4	4698	0.0013
hsa-miR-301a-3p	5168.47	22	52418	0.0013
hsa_circRNA_104682	4380.00	2	16344	0.0013
YOD1	4380.00	2	160	0.0010
hsa-miR-196a-5p	4298.00	15	14042	0.0009
CCND2	4280.22	2	1440	0.0014
hsa-miR-652-5p	4166.00	4	7722	0.0011

## Supplementary Materials

Supplementary Materials Table S1: differentially expressed circRNAs between tumor and normal tissues. Table S2: differentially expressed miRNAs between tumor and normal tissues.
